# Unveiling Stimulation Secrets of Electrical Excitation of Neural Tissue Using a Circuit Probability Theory

**DOI:** 10.3389/fncom.2020.00050

**Published:** 2020-07-10

**Authors:** Hao Wang, Jiahui Wang, Xin Yuan Thow, Sanghoon Lee, Wendy Yen Xian Peh, Kian Ann Ng, Tianyiyi He, Nitish V. Thakor, Chengkuo Lee

**Affiliations:** ^1^Institute of Biomedical and Health Engineering, Shenzhen Institutes of Advanced Technology (SIAT), Chinese Academy of Sciences (CAS), Shenzhen, China; ^2^Department of Electrical and Computer Engineering, National University of Singapore, Singapore, Singapore; ^3^Center for Intelligent Sensor and MEMS, National University of Singapore, Singapore, Singapore; ^4^Hybrid Integrated Flexible Electronic Systems, National University of Singapore, Singapore, Singapore; ^5^Singapore Institute for Neurotechnology (SINAPSE), National University of Singapore, Singapore, Singapore; ^6^Department of Robotics Engineering, Daegu Geongbuk Institute of Science and Technology (DGIST), Daegu, South Korea; ^7^NUS Graduate School for Integrative Sciences and Engineering, National University of Singapore, Singapore, Singapore

**Keywords:** electric nerve stimulation, mathematical model, circuit-probability theory, computational modeling, inductor in neural circuit

## Abstract

Electrical excitation of neural tissue has wide applications, but how electrical stimulation interacts with neural tissue remains to be elucidated. Here, we propose a new theory, named the Circuit-Probability theory, to reveal how this physical interaction happen. The relation between the electrical stimulation input and the neural response can be theoretically calculated. We show that many empirical models, including strength-duration relationship and linear-non-linear-Poisson model, can be theoretically explained, derived, and amended using our theory. Furthermore, this theory can explain the complex non-linear and resonant phenomena and fit *in vivo* experiment data. In this letter, we validated an entirely new framework to study electrical stimulation on neural tissue, which is to simulate voltage waveforms using a parallel RLC circuit first, and then calculate the excitation probability stochastically.

## Introduction

Neuromodulation by electrical stimulation has proven itself as an effective treatment for medical conditions in many therapeutic situations, including deep brain stimulation [e.g., Parkinson's disease; Shah et al., 2017], spinal cord stimulation (e.g., chronic pain) and peripheral nerves stimulation (neuroprosthetics) (Berger et al., 2011; Gilja et al., 2012). Despite these wide applications, fine details of the mechanism by which electrical stimulation modulates neural response remains elusive (Howell et al., 2015; Pelot et al., 2017), and a more complete theoretical model accounting for tissue response to various electrical stimulation parameters is still an ongoing pursuit (Keener, 1996; Pumir and Krinsky, 1996; Ma et al., 2010; Brocker and Grill, 2013; Fertonani and Miniussi, 2017). The conventional approach is to study stimulation of individual neurons based on the Hodgkin–Huxley model (HH model) along with simulated electric field (E-field) distribution (Hodgkin and Huxley, 1952; Raspopovic et al., 2011; Capogrosso et al., 2013; Pelot et al., 2017). However, there is a gap in our knowledge describing the microscopic axon structure leading up to the stimulation and response in complex neural (nerve and cortex) and non-neural (muscle) tissues (Morse et al., 2015). To address this issue, empirical models and rules have been developed [e.g., Linear-Nonlinear-Poisson cascade model (LNP model) (Schwartz et al., 2006), strength-duration curves (Lapicque, 1909; Weiss, 1990), and stimulation waveforms efficiency difference Offner, 1946; Yuen et al., 1981; McCreery et al., 1992; Shannon, 1992; Jezernik and Morari, 2005; Wongsarnpigoon and Grill, 2010]. Still, some important phenomena, such as the frequency dependent response of nerve fibers (Evans, 1972; Li and Bak, 1976; Hartmann et al., 1984; Kral et al., 1998; Howell et al., 2015) and the stochastically distributed gating pattern of the ion channels (Sanchez et al., 1986; Aldrich and Stevens, 1987; Bezanilla, 2000; White et al., 2000), remain unaccounted for.

Here, we propose a new theory, named Circuit-Probability (C-P) theory, to provide a physical framework, which is completely different from the conventional way of using H-H model with E-field modeling. Then, we show that some widely-used empirical models and rules can be intuitively derived from the C-P theory.

## Method to Obtain the Circuit-Probability Framework

How should we analyze tissue response to an external stimulation? To answer this question, we performed a thought experiment, which ultimately led to our new framework of Circuit-Probability. When considering the electrode-tissue interaction, the first question is how the electrode is bridged to the tissue. We know the activation of action potential is induced by the gating of the voltage-dependent ion channels. Then, for electrode-tissue interaction, the key issue is how the electrical input affects the voltage on these ion channels. Considering the cell membrane is a capacitor, which is impermeable to ions, it affects its electrical response in two aspects. Firstly, the voltage changes on the capacitor, which is induced by charging and discharging procedures, will generate a different waveform in response to the input waveform. And the charging and discharging procedures are not only affected by the capacitor itself, but also affected by its peripheral circuit. Secondly, the E-field will always be perpendicular to the plate of the capacitor, which is the cell membrane surface, and the direction of the E-field is only determined by the orientation of the capacitor. Apparently, the correct voltage waveform and correct E-field direction can be both obtained with a proper circuit involving the capacitor of cell membrane. This is why we use a circuit to characterize the electric response on the cell membrane.

With a proper circuit, we can model the voltage waveform. Then, from this voltage waveform, how can we know the stimulation strength? In the *in vivo* testing, the number of activated action potentials shows a continuous change with the electric input. However, single channel measurement shows that an individual ion channel does not display a continuous state change in response to electric input. It acts like a digital bit, which only has two states: closed and open. Meanwhile, the gating pattern of a single ion channel also shows a stochastic behavior. Then, how can we build a bridge from the microscopic discrete to macroscopic continuity? The exclusive option is a probabilistic description of the ion channel gating, just as the situation of thermal dynamics and quantum mechanics. The ion channel gating is stochastic and can be described by the exponential distribution. When a specific electrical field is applied onto the ion channel, the time duration it takes to open the ion channel can be described by the exponential distribution. Then with the voltage waveform simulated using the circuit, it is easy to calculate the probability of activating an action potential with a certain electrical input.

Up to here, we have obtained a basic framework of Circuit-Probability based on pure physical reasoning. The proper circuit configuration can only be fitted using experimental results, which is a posteriori, while the probability equation can obtained by theoretical derivation, which is a priori.

## The Method to Obtain the Probability Calculus in Circuit-Probability Theory

Here we firstly show how to theoretically derive the probability equation.

In the electrical stimulation of a neuron, we assume that electron transition of the protein causes the opening of sodium ion channel, which then generates an action potential (AP). Electron transition is a quantum phenomenon, which is random. Hence, the generation of APs can be described with an exponential distribution for quantum event:

(1) $$\begin{array}{r} \text{f}\left(\lambda ,\text{t}\right)=\{\begin{array}{cc} 1-{\text{e}}^{-\lambda \text{t}} & ,\;\text{t}\geq 0\\ 0 & ,\;\text{t}<0 \end{array}\;\lambda >0 \end{array}$$

Here *f*(λ, *t*) represents the probability of AP to be generated within a time duration of *t*. $\frac{1}{\lambda }$ is the expected time until AP is generated. Expand the exponential distribution to a general calculus form:

(2) $$\begin{array}{r} \text{f}\left(\lambda ,\text{t}\right)=\;1-{\text{e}}^{-\int \lambda \left(\text{t}\right)\text{d}t} \end{array}$$

then the normal exponential distribution is the special form when λ(*t*) is a constant.

Meanwhile, $\frac{1}{\lambda }$ is also a function of the voltage *V*:

(3) $$\begin{array}{r} \frac{1}{\lambda }=\text{g}(\text{V}) \end{array}$$

We have three electrophysiological considerations for *g*(*V*):

**Consideration 1**: $\frac{1}{\lambda }$ to be infinitely large when the voltage, *V*, is more positive than the threshold voltage, *V*_*Threshold*_. In this condition, the AP cannot be generated.
**Consideration 2**: $\frac{1}{\lambda }$ to be inversely proportional with the amplitude of |*V* − *V*_*Threshold*_|, when *V* is more negative than *V*_*Threshold*_.
**Consideration 3**: $\frac{1}{\lambda }$ to approach a minimal level when |*V* − *V*_*Threshold*_| goes to infinite. So $\frac{1}{\lambda }$ should get saturated at a certain value.

With these three considerations, one possible form of *g*(*V*) can be expressed as:

(4) $$\frac{1}{\lambda }=g\left(V\right)=\frac{1}{\alpha }\times (e^{\frac{\beta }{{\left|V-V_{Threshold}\right|}^{n}}}-c)$$

The equation can be re-written as:

(5) $$\begin{array}{r} \lambda =\frac{1}{g\left(V\right)}=\alpha \times \frac{1}{e^{\frac{\beta }{{\left(\left|V-V_{Threshold}\right|\right)}^{n}}}-c} \end{array}$$

Here, α, β, *n*, and *c* are adjustment parameters, where α > 0, β > 0, *n* > 1, and 0 ≤ *c* ≤ 1.

To simplify the equation, here we assume that *n* = 1 and *c* = 0.

Then the complete expression of λ is:

(6) $$\begin{array}{r} \lambda =\{\begin{array}{cc} \alpha \times e^{-\frac{\beta }{\left|V-V_{Threshold}\right|}},\;V<V_{Threshold}\\ 0\;, & V\geq V_{Threshold} \end{array} \end{array}$$

Considering the voltage waveform, *V*(*t*), is a function of time, *t*, the complete probability calculus equation is:

(7) $$\begin{array}{r} f\left(\lambda ,t\right)=\;1-e^{-\int \lambda \left(t\right)dt}=1-e^{-\int \lambda \left(V\left(t\right)\right)dt}\\ =1-e^{-\alpha \int e^{-\frac{\beta }{\left|V\left(t\right)-V_{Threshold}\right|}}dt},\;V\left(t\right)<V_{Threshold} \end{array}$$

In this equation, α, β, and *V*_*Threshold*_ are three parameters to be determined by data fitting.

**Figure 1:**
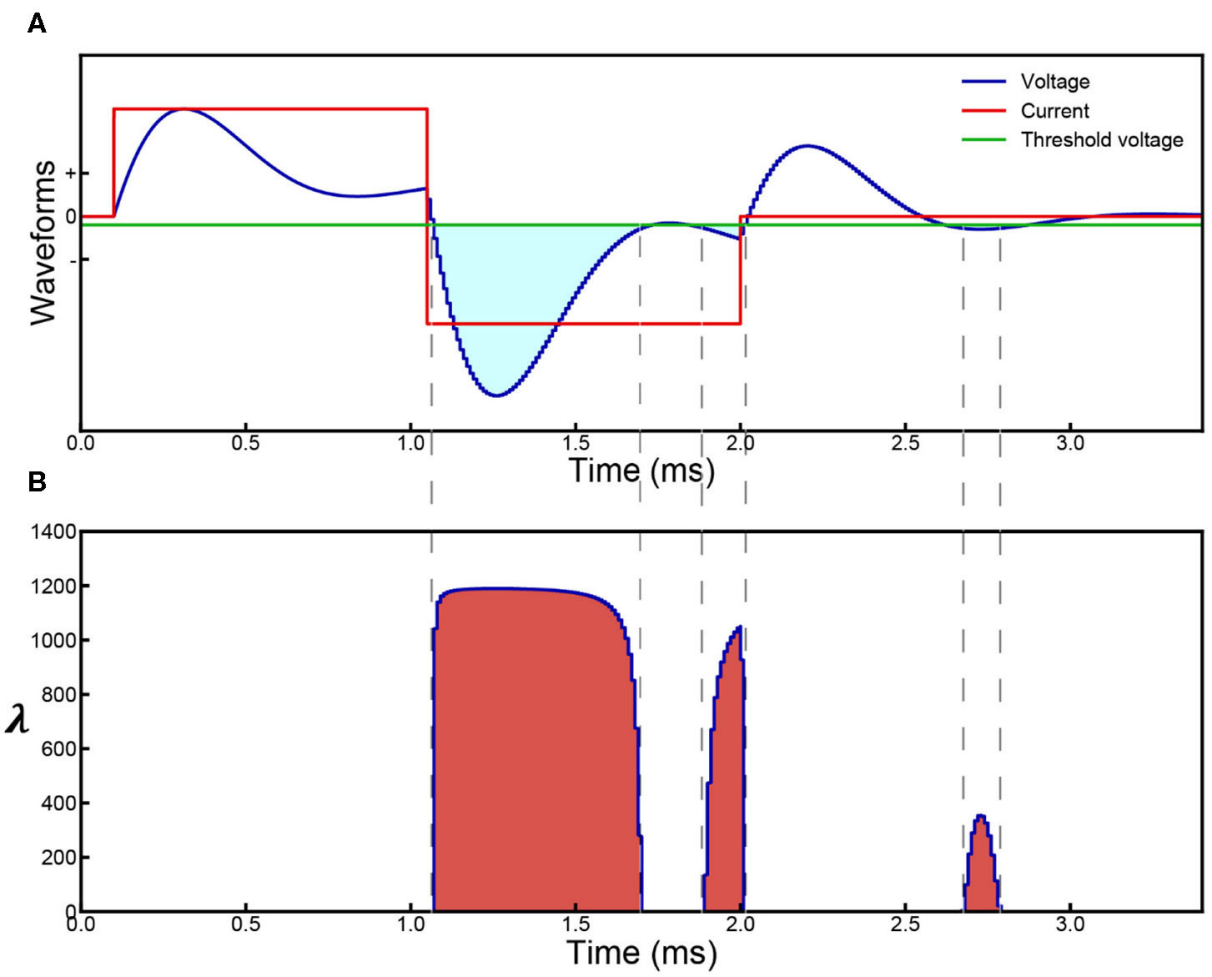
Parameter illustration of the probability calculus. **(A)** An illustrative case with multiple effective voltage areas. Red line represents the input current (biphasic in this case), blue line represents the resultant voltage across the cell membrane, and the green line represents the threshold voltage for action potential generation. **(B)** Corresponding λ curve converted from the voltage curve in **(A)**. The probability, *P*, will change monotonically with the area of the λ curve, *S*_λ_.

For a specific voltage waveform as shown in Figure 1A, the voltage waveform can be converted to a λ waveform as shown in Figure 1B. Then the probability calculus can be further simplified as:

(8) $$\begin{array}{r} f=1-e^{-\int \lambda \left(t\right)dt}=1-e^{{-S}_{\lambda }} \end{array}$$

where *S*_λ_ is the area of the λ waveform. A detailed analysis of the probability calculus can be found in Supplementary S1.

## Method to Obtain a Correct Neural Circuit in Circuit-Probability Theory

**Figure 2:**
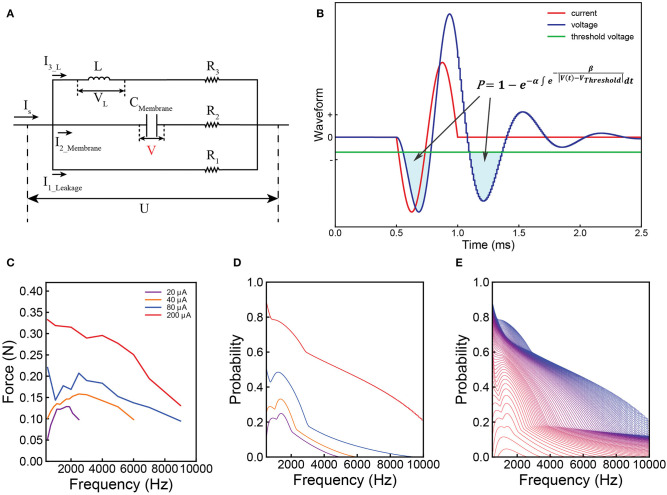
Illustration of the Circuit-Probability (C-P) theory with experiment and modeling results from the Common Peroneal (CP) nerve stimulation with sine-wave current. **(A)** The equivalent parallel RLC circuit of the neural tissue; **(B)** a graph of the applied current (red line) and resulting voltage (blue line) waveforms produced across the capacitor of the circuit as shown in **(A)**; This response is derived from the probability calculus in equation: $P=\;1-e^{-\alpha \int e^{-\frac{\beta }{\left|V\left(t\right)-V_{Threshold}\right|}}dt}$; **(C)** the force mapping result recorded from the TA muscle by nerve stimulation. Four different current amplitudes were used at different frequencies, spanning from 500–9,000 Hz; **(D)** the corresponding modeling results showing the local minima and maxima predicted by the C-P theory; **(E)** a detailed probability mapping showing how the shape of the probability curve changes from low current, which exhibits the resonance effect, to high current, which has monotonically decreasing trend.

Then, we build a proper circuit using the results shown in Figure 2. Its general configuration and analysis can be obtained by reasoning (Supplementary S2). Based on the general configuration, its exact configuration is obtained by fitting the experiment data. This is a parallel RLC circuit. The capacitor refers to the cell membrane. The inductor is included to explain the frequency dependent response observed in the experiments. We validated this by applying a single-frequency input (sinusoid wave) to the Common Peroneal (CP) nerve. Sine wave currents (red curve in Figure 2B) were applied upon the CP nerve to activate the Tibialis Anterior (TA) muscle and the resulting force was recorded. The force measured with respect to frequency forms a curve, here named as ‘force mapping curve' in this study. With a specific current waveform, a resultant voltage waveform on the capacitor can be calculated, shown as the blue curve in Figure 2B for probability calculation. Similarly, a probability curve with respect to frequency calculated by modeling is defined as a probability mapping curve. The detailed experiment procedure and testing setup can be found in the Supplementary S3. The force mapping results (force generated by TA muscle) against the pulse width of single pulses (in Hz) of four different current amplitudes curves are shown in Figure 2C. The same data plotted with error bar can be found in the Supplementary S6.1.

The shapes of these four curves are quite different, showing a complicated changing trend with increasing current amplitude. For the curves of 20 and 40 μA, a clear resonance effect can be observed. However, 80 μA curve shows an initial decline, before increasing to a resonance frequency. The curve of 200 μA shows a monotonically decreasing trend without the resonance effect. Despite these variations, C-P theory can still reproduce the general shapes of the curves via probability mapping (Figures 2D,E shows a more detailed probability mapping). The parameters for the circuit and probability calculus can be found in Table 1-1(d&e). It clearly shows how the force-frequency curve changes from one shape to another shape with increasing current amplitude over a variety of pulse frequencies and accurately predicts the trend, particularly the existence of local minima and maxima. The probability mapping from the C-P theory reproduces the complex changing trends of the testing results, validating the parallel RLC circuit, probability calculus, and most importantly, the existence of an inductor.

**Table 1:** Modeling parameters.

**No**	**R_1_(Ω)**	**R_2_(Ω)**	**R_3_(Ω)**	**C(F)**	**L(H)**	****α****	****β****	**V_Threshold_(V)**
1 (d&e)	3,45,000	5,000	10,000	9n	1.9545	2,000	0.1	−0.6
3 (b,c)	16,579	100	3,000	12n	2.1109	NA	NA	From −0.09 to −0.17
S4.1 (b)	16,579	100	3,000	12n	2.1109	1,200	0.01	−0.08
S4.2 (a)	11,052	100	3,000	12n	0.5277	2,000	0.04	−0.048
S5 (a-i)	5,181	100	200	12n	0.1938	2,000	0.1	−0.1
S5 (a-ii)	5,181	100	2,000	12n	0.1938	2,000	0.1	−0.1
S5 (b-i)	10,362	100	200	12n	0.1938	2,000	0.1	−0.1
S5 (b-ii)	10,362	100	2,000	12n	0.1938	2,000	0.1	−0.1
S5 (c-i)	20,723	100	200	12n	0.1938	2,000	0.1	−0.1
S5 (c-ii)	20,723	100	2,000	12n	0.1938	2,000	0.1	−0.1
S6.2.1 (a)	80,000	300	1,700	18n	0.1086	NA	NA	NA
S6.2.1 (b)	2,656	1,800	800	18n	0.0813	NA	NA	NA
S6.2.1 (c)	2,656	1,800	800	18n	0.0813	NA	NA	NA
S6.2.1 (d)	2,000	1,350	500	10n	0.1464	NA	NA	NA
S6.2.1 (e)	3,701	350	500	10n	0.1464	NA	NA	NA
S6.2.1 (f)	9,000	1,350	500	10n	0.2326	NA	NA	NA
S6.3.1.1 (b)	60,286	1,800	2,000	4n	5.2335	600	0.8	−0.7
S6.3.1.2 (b)	72,343	4,600	14,400	4n	5.2335	1,500	0.06	−9.69
S6.3.1.3 (b)	100	100	300	100n	0.1621	45,000	0.0075	−0.006
S6.4.1 (b)	12,384	1,200	18,000	10n	4.9687	13,000	0.5	−0.35
S6.4.2 (b)	5,000	30	200	C1 = 400n; C2 = 5,000n	0.0702	2,000	0.015	−0.009
S6.5.1 (b)	90,000	100	600	12n	0.1629	17,000	0.58	−0.22

## Simulation Fitting to Experimental Results by Circuit-Probability Theory

The C-P framework and the probability calculus equation is achieved by reasoning, which is a priori, rather than a posteriori. This is very unusual for biological research. Meanwhile, the circuit is still of a preliminary configuration. To validate the correctness of this priori theory, a series of experiments on four types of non-neural and neural tissues using a rat model were conducted: the skeletal muscles (Supplementary S6.3), the sciatic nerve (Supplementary S6.4), the cortex (Supplementary S6.5), and the pelvic nerve (Supplementary S6.6). All the testing data can be well-fitted or explained by the C-P theory: 1. Different current waveforms will generate force mapping curves with different shapes; 2. Force mapping curves generated by arbitrary current waveforms can be fitted by modeling of C-P theory; 3. The resonance frequency widely exists in nervous systems and can be measured with proper stimulation parameters. To help readers understand how various force mapping patterns are generated and affected by parameters, a general demonstration (Supplementary S5) and a detailed case analysis (Supplementary S4) of how the circuit parameters affect the probability mapping pattern are provided.

Meanwhile, C-P theory can give a unique prediction: the electrical voltage response by electrical stimulation, which is conventionally considered as the stimulus artifact, can be well-fitted by the voltage response of the circuit in Figure 2A. This voltage response will show the same voltage response as a parallel RLC circuit. The data by experiment and modeling can be found in Supplementary S6.2.

## Theoretical Explanation to Strength-Duration Relationship and LNP Model by Circuit-Probability Theory

This C-P theory provides a physical understanding of the electrical nerve stimulation, which is not available in previous theories and models. Thus, most of the phenomenological models and theories can be directly derived or even amended from C-P theory. Here we just show how to derive and correct two well-known phenomenological models in electrical nerve stimulation: strength-duration relationship (Lapicque, 1909; Weiss, 1990) and LNP model (Linear-Nonlinear-Poisson cascade model) (Schwartz et al., 2006).

Firstly, we will derive and amend the strength-duration relationship. Previously, it is widely believed that charge is the factor to induce nerve stimulation. In such charge based theory, there is an empirical linear relationship between the threshold charge level and pulse duration, which is called Weiss's strength–duration equation (Weiss, 1990) for negative monophasic square current pulses. This equation describes the threshold charge as a function of pulse width as follows:

(9) $$\begin{array}{r} Q_{th}\left(PW\right)=I_{rh}\times PW+T_{ch}\times I_{rh} \end{array}$$

where *I*_*rh*_ is the rheobase current, *T*_*ch*_ is the chronaxie, and *PW* is the pulse width. The rheobase current is defined as the threshold current for infinitely long pulses. The chronaxie is defined as the pulse duration required for excitation when the current amplitude is equal to twice the rheobase current. And Lapicque reiterated Weiss's equation for the strength–duration relationship (Lapicque, 1909), but in terms of the threshold current, and introduced the rheobase current and chronaxie as the constants:

(10) $$\begin{array}{r} I_{th}\left(PW\right)=I_{rh}\left(1+\frac{T_{ch}}{PW}\right) \end{array}$$

Apparently, these two equations are just mathematical descriptions without explaining how *I*_*rh*_ happen and why the curve follows a specific trend.

As follows is the derivation of this relationship with physical definition of *I*_*rh*_.

**Figure 3:**
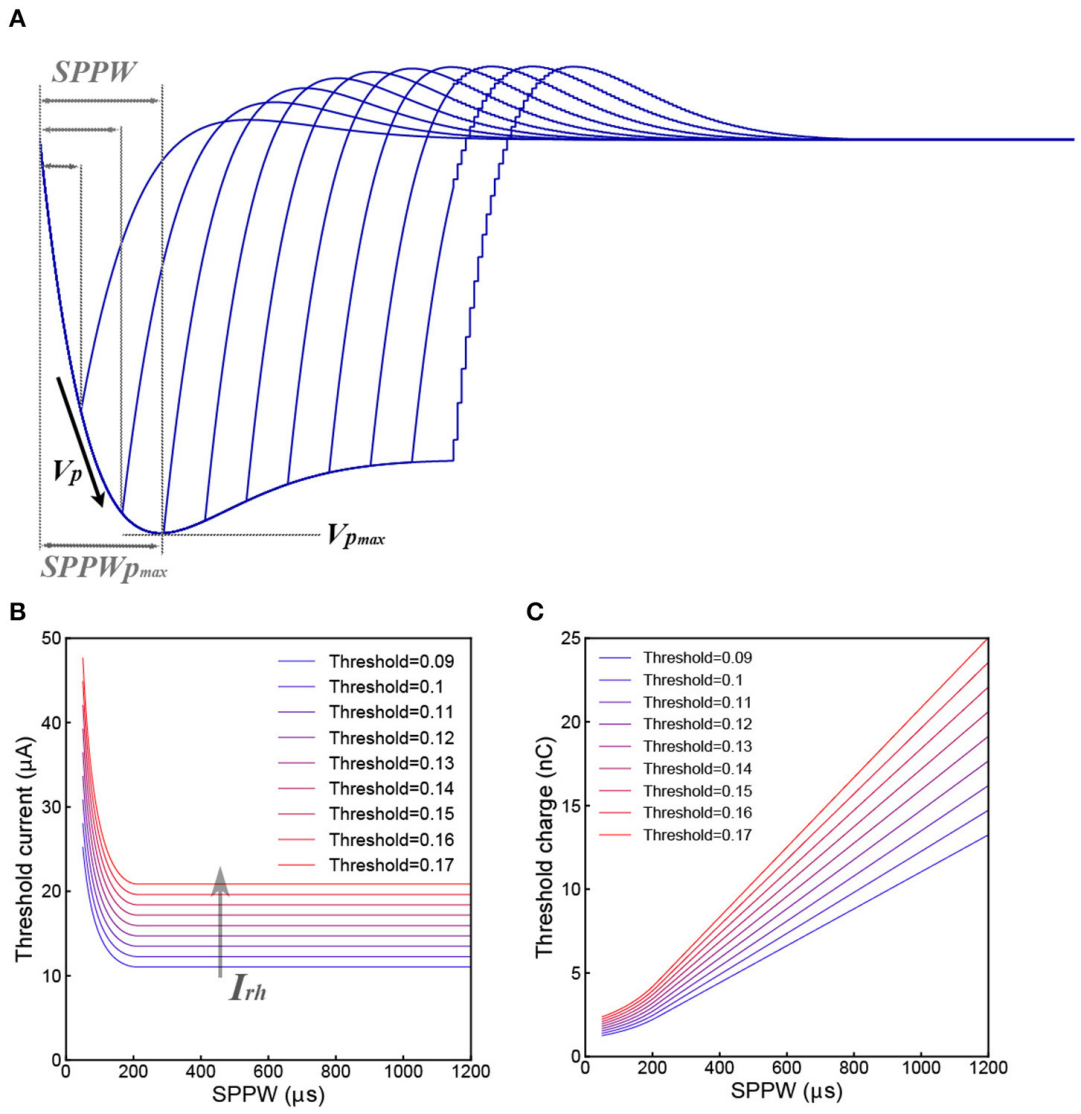
Derivation of the Strength–duration relationship. **(A)** Illustrative voltage waveforms generated by negative monophasic square waveform current; **(B)** the threshold current amplitude (*I*_*th*_) decreases as the *SPPW* increases in a non-linear fashion; **(C)** the relationship between threshold charge (*Q*_*th*_) and *SPPW* is linear.

Figure 3A shows a typical voltage waveform by applying negative monophasic square current with difference SPPW (single phase pulse width). For the voltage waveform of each SPPW, the peak voltage is denoted as *V*_*P*_, which is a function of *I* and *SPPW* and written as *V*_*P*_(*I, SPPW*). Based on C-P theory, nerve excitation can be induced when *V*_*P*_(*I, SPPW*) ≥ *V*_*Threshold*_. Then both the threshold current *I*_*th*_ and the threshold charge, *Q*_*th*_ = *I*_*th*_ × *SPPW*, are defined as the current and charge required to make the *V*_*P*_ reaches *V*_*Threshold*_.

Then the critical condition is:

(11) $$\begin{array}{r} V_{P}\left(I_{th},SPPW\right)=V_{Threshold} \end{array}$$

*I*_*th*_ and *Q*_*th*_ can be written as functions of *SPPW* and *V*_*Threshold*_:

(12) $$\begin{array}{r} I_{th}=f\left(SPPW,V_{Threshold}\right) \end{array}$$

(13) $$\begin{array}{r} Q_{th}=I_{th}\times SPPW=f\left(SPPW,V_{Threshold}\right)\times SPPW \end{array}$$

Since *V*_*P*_(*I*_*th*_, *SPPW*) cannot be expressed analytically, only numerical solutions of *I*_*th*_ and *Q*_*th*_, which are calculated with a set of modeling parameter [Table 1-3(b,c)] are provided in Figures 3B,C. In Figure 3B, all curves decrease to a constant value, *I*_*rh*_. This is because the *V*_*P*_ will saturate at a maximum value, *V*_*P*_max__, when *SPPW* ≥ *SPPW*_*P*_max__, as shown in Figure 3A.

Meanwhile,

(14) $$\begin{array}{r} Q_{th}=I_{th}\times SPPW=I_{rh}\times SPPW\;when\;SPPW\geq SPPW_{P_{\max}} \end{array}$$

Since *I*_*rh*_ is a constant, *Q*_*th*_ increases linearly with *SPPW*, when *SPPW* ≥ *SPPW*_*P*_max__, as shown in Figure 3C.

The physical meaning of *I*_*rh*_ is the threshold current when *V*_*P*_max__ = *V*_*Threshold*_. Meanwhile, the non-linear curve of *I*_*th*_ vs. *SPPW*, existence of *I*_*rh*_ and linear curve of *Q*_*th*_ vs. *SPPW*, can be directly obtained without any additional hypotheses. The exact analytical equation for this relationship is not available. The curves in Figures 3B,C are the numerical solution of strength–duration relationship. It corrects the relationship in two aspects:

1. Rather than infinitely approaching to the *I*_*rh*_ as the case in Weiss's strength–duration equation, the threshold current curve will be equal to the *I*_*rh*_ when *SPPW* ≥ *SPPW*_*P*_max__.
2. Rather than being a completely straight line, the threshold charge curve is linear only when *SPPW* ≥ *SPPW*_*P*_max__. When the *SPPW* is approaching zero, the slope of threshold charge curve will also approach zero, meaning that the threshold charge will converge in a constant value at low *SPPW*.

These two major special differences with the Weiss's equation have already be confirmed by previous research (Friedli and Meyer, 1984; Su et al., 2017) and now can be well-explained in the C-P theory.

**Figure 4:**
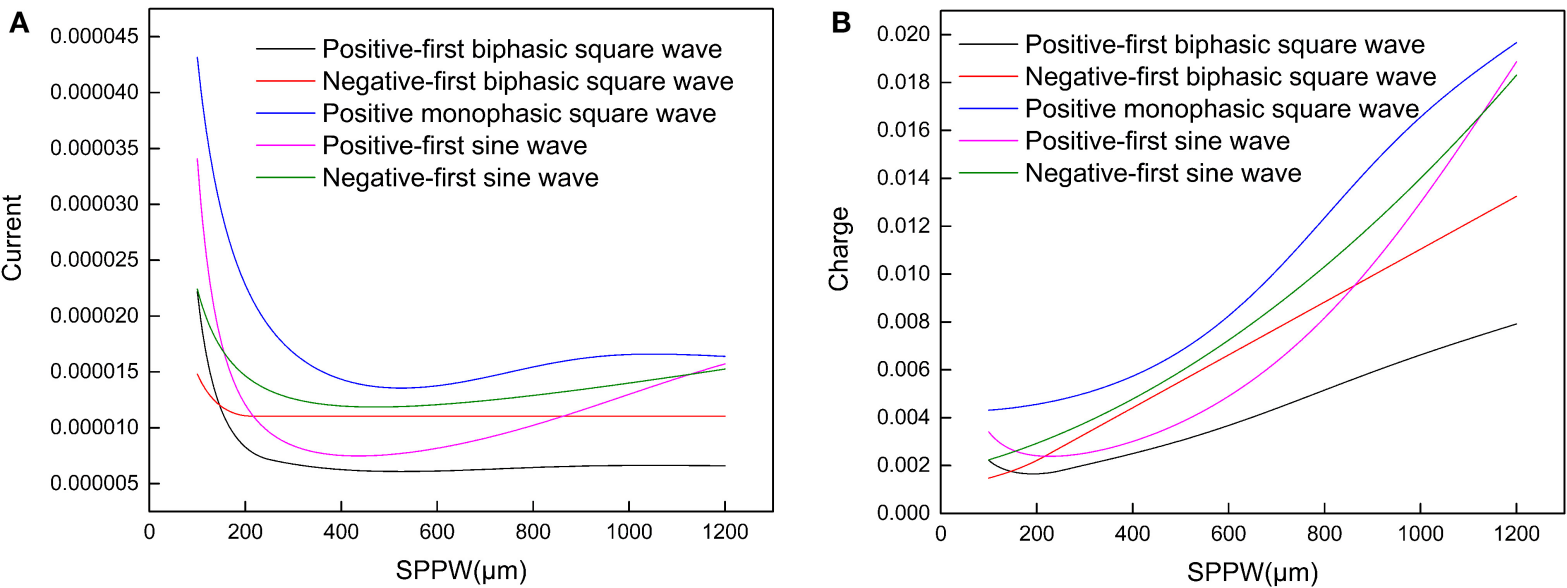
**(A)** The relationship between the threshold current amplitude (*I*_*th*_) and the *SPPW* for different current waveforms; **(B)** the relationship between threshold charge (*Q*_*th*_) and *SPPW* for different current waveforms.

Moreover, it also explains why such relationship can only be applied for negative monophasic square current waveform. Because the voltage waveforms differs with the current waveforms, inducing a more complicated trend without a stable *I*_*rh*_, which was observed in other researches (Friedli and Meyer, 1984). In Figure 4, representative strength–duration curves of other waveforms including different types of square waves and sine waves are shown. For the curve of sinewave current, the threshold current curve increases at high SPPW range, this phenomenon has been observed in previous research with triangle current waveform (Rodríguez-Fernández et al., 2016). But these curves also vary with different circuit parameters.

Then, we will derive the LNP model. The LNP model is a simplified functional model of neural spike responses (Schwartz et al., 2006). It has been successfully used to describe the response characteristics of neurons in early sensory pathways, especially the visual system. The LNP model is generally implicit when using reverse correlation or the spike-triggered average to characterize neural responses with white-noise stimuli. The number of action potential generated can be described by the Poisson distribution in LNP model.

Actually, the Poisson distribution and exponential distribution describe the same stochastic process. If the Poisson distribution provides an appropriate description of the number of the occurrences per interval of time, then the exponential distribution will provide a description of the time interval between occurrences.

The Poisson distribution is as follow:

(15) $$\begin{array}{r} P\left(x=k;\lambda \right)=\frac{\lambda^{k}}{k!}e^{-\lambda } \end{array}$$

*P*(*x* = *k*; λ) is the probability of the *k* times occurrences of the event in a unit time interval, **λ** is the expected times of occurrence.

The exponential distribution is as follow:

(16) $$\begin{array}{r} P\left(t;\lambda \right)=1-e^{-\lambda t} \end{array}$$

*P*(*t*; λ) is the probability of the occurrences of the event with the time interval *t*, $\frac{1}{\lambda }\;$is the expected time interval.

These two distributions share the same λ. Apparently, in the C-P theory, if the generation of action potential can be described by exponential distribution, it surely can be described by Poisson distribution.

As follow is the derivation of LNP model.

**Figure 5:**
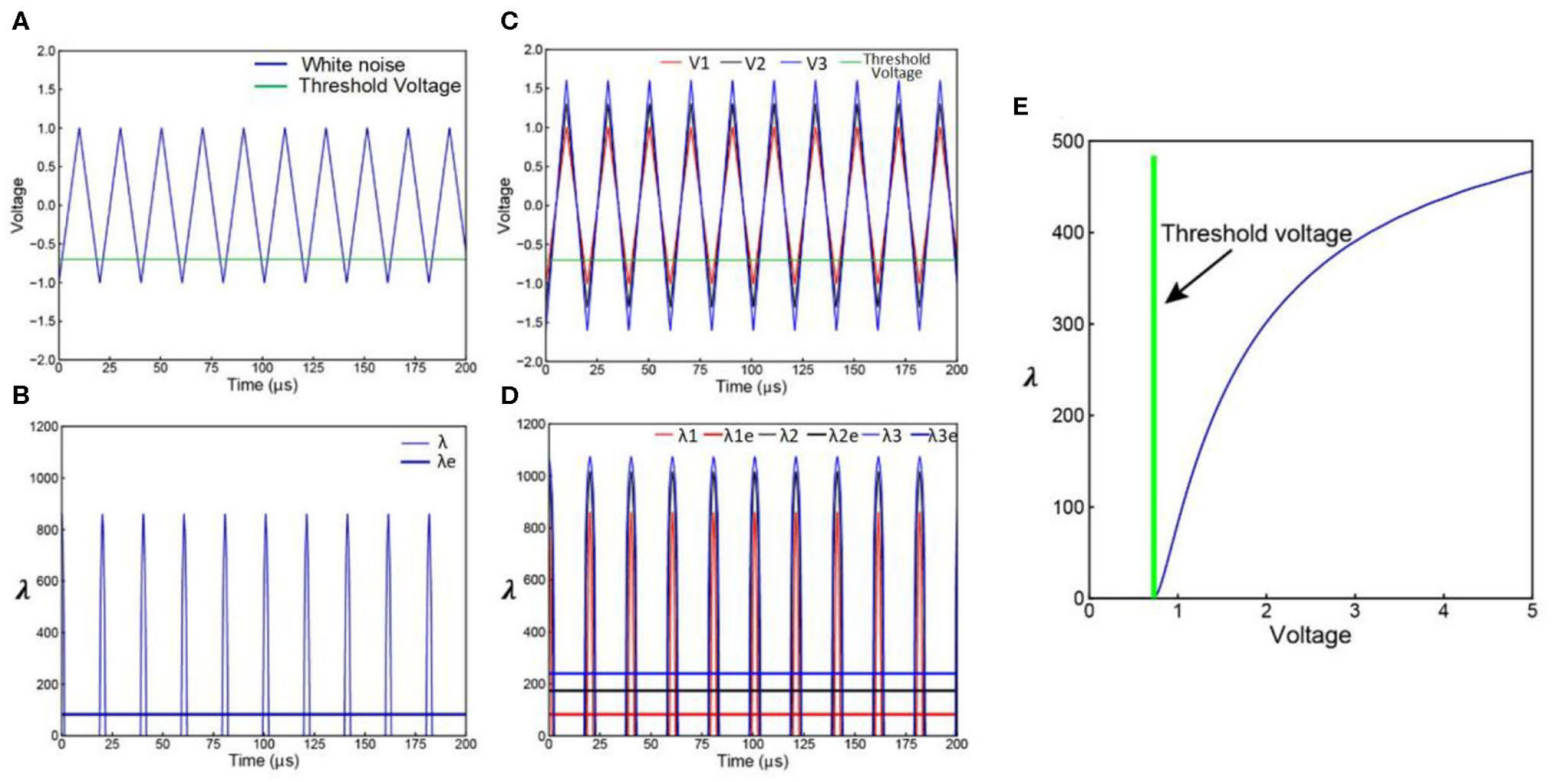
Derivation of LNP model from C-P theory. **(A)** A simplified white noise voltage waveform; **(B)** the corresponding λ curve of the voltage waveform in **(A)**, this λ curve can be averaged to a λ_*e*_ curve; **(C)** noise with increasing amplitude; **(D)** the corresponding λ and λ_*e*_curves of the noise waveforms in **(C)**; **(E)** the non-linear curve of λ_*e*_ vs. the noise amplitude *V*_*w*_.

The white noise involved in LNP model can be simplified as a triangle wave series of frequency *f* and amplitude *V*_*w*_ as shown in Figure 5A. Actually, any kind of periodical voltage waveform can be used. The triangle wave is used as an example of simple waveform.

Only part of the voltage can exceed the *V*_*Threshold*_. As explained in Figure 1B, the voltage curve can be converted to a λ curve as shown in Figure 5B. The area *S*_λ_ of the λ curve within a period $T=\frac{1}{f}$ can be calculated. Since the λ implemented in the C-P theory is not a constant value while λ in Poisson distribution can only be a constant value, an equivalent λ_*e*_ for Poisson distribution can be calculated based on the *S*_λ_:

(17) $$\begin{array}{r} \lambda_{e}=\frac{S_{\lambda }}{T}=S_{\lambda }\times f \end{array}$$

which is the blue straight line in Figure 5B. Apparently, the λ curve and the λ_*e*_ curve are of the same area, so they will induce the same statistical results.

So the probability calculus equation can be rewritten as:

(18) $$\begin{array}{r} P=1-e^{{-S}_{\lambda }}=1-e^{-\lambda_{e}t} \end{array}$$

The corresponding Poisson distribution is:

(19) $$\begin{array}{r} P\left(x=k;\lambda_{e}\right)=\frac{{\lambda_{e}}^{k}}{k!}e^{-\lambda_{e}} \end{array}$$

By increasing the noise amplitude *V*_*w*_, *S*_λ_ will also increase, result in an increasing **λ**_**e**_ as shown in Figures 5C,D. Since *S*_λ_ is a function of *V*_*w*_, and λ_*e*_ is a function of *S*_λ_, λ_*e*_ is also a function of *V*_*w*_, shown as the non-linear curve in Figure 5E. This explains how a linear increment of *V*_*w*_ induces a non-linear increment of λ_*e*_ happened in LNP model. Because the expression of *S*_λ_ is a piecewise function of *V*_*w*_, the exact function λ_*e*_(*V*_*w*_) can only be calculated numerically with a fixed α, β, *V*_*Threshold*_, and *f*. The analytical expression of λ_*e*_(*V*_*w*_) is not available.

## Summary

In summary, we propose a new theory, named the Circuit-Probability theory, to unveil the “secret” of electrical nerve stimulation, essentially explain the non-linear and resonant phenomena observed when nerves are electrically stimulated. In this theory, an inductor is involved in the neural circuit model for the explanation of frequency dependent response. Furthermore, predicted response to varied stimulation strength is calculated stochastically. Two empirical models, strength-duration relationship and LNP model, can be theoretically derived from C-P theory. This theory is shown to explain the complex non-linear interactions in electrical nerve stimulation and fit *in vivo* experiment data on stimulation-responses of many nerve experiments. As such, the C-P theory should be able to guide novel experiments and more importantly, offer an in-depth physical understanding of the neural tissue. More detailed discussion about possible issues, such as the inductance and probability calculus, can be found in Supplementary S7. As a promising neural theory, we can even further explore the more accurate circuit configuration and probability equation to better describe the electrical stimulation of neural tissues in the future.

## Data Availability Statement

All datasets generated for this study are included in the article/Supplementary Material.

## Ethics Statement

This animal study was reviewed and approved by IACUC of National University of Singapore.

## Author Contributions

The theory was developed by HW, JW, and TH. The modeling work was carried out by HW. The major framework of experiment design was carried out by HW, JW, and XT. The experiments of CP nerve stimulation were carried out by SL, JW, XT, and HW. The experiments of TA muscle stimulation were carried out by JW, XT, and HW. The experiments of cortical stimulation were carried out by XT, JW, and HW. The experiments of pelvic stimulation were carried out WP, JW, and HW. The experiments of stimulus artifact recording with high sampling frequency system were carried out by KN, XT, JW, and HW. The data analysis was carried out by HW, JW, XT, and WP. The manuscript was written by HW, JW, and XT. NT and CL provided general guidance of the project. All authors discussed the experimental results and contributed to the final version of the manuscript.

## Conflict of Interest

The authors declare that the research was conducted in the absence of any commercial or financial relationships that could be construed as a potential conflict of interest.
